# Constructive Autoassociative Neural Network for Facial Recognition

**DOI:** 10.1371/journal.pone.0115967

**Published:** 2014-12-26

**Authors:** Bruno J. T. Fernandes, George D. C. Cavalcanti, Tsang I. Ren

**Affiliations:** 1 Escola Politécnica, Universidade de Pernambuco, Recife-PE, Brazil; 2 Centro de Informática, Universidade Federal de Pernambuco, Recife-PE, Brazil; Georgia State University, United States of America

## Abstract

Autoassociative artificial neural networks have been used in many different computer vision applications. However, it is difficult to define the most suitable neural network architecture because this definition is based on previous knowledge and depends on the problem domain. To address this problem, we propose a constructive autoassociative neural network called CANet (Constructive Autoassociative Neural Network). CANet integrates the concepts of receptive fields and autoassociative memory in a dynamic architecture that changes the configuration of the receptive fields by adding new neurons in the hidden layer, while a pruning algorithm removes neurons from the output layer. Neurons in the CANet output layer present lateral inhibitory connections that improve the recognition rate. Experiments in face recognition and facial expression recognition show that the CANet outperforms other methods presented in the literature.

## Introduction

Computer models developed based on theories of the human brain structure have been applied in various problems in computer vision. While the human brain has not been fully well understood, these models inspired many methods used in pattern recognition, such as Artificial Neural Networks (ANNs) [1]. ANNs have been improved using concepts based on theories of the human brain such as the receptive and inhibitory fields (also known as lateral inhibition) [2]–[5] and the autoassociative memory [6]. Receptive fields concepts have been used in neural networks for implicit feature extraction of input images, where neurons are connected to predefined regions in previous layers [7]–[10]. The lateral inhibition concepts are applied in order to improve the neural network stability and efficiency, making the model less sensitive to image distortions [11]–[14]. Autoassociative memory inspired the development of neural networks for one-class classification tasks, enabling the models to be suitable for learning with only positive patterns. Consequently, this establish closed decision boundaries in the input space.

In previous works, we proposed two neural networks for visual pattern classification: LIPNet [7] and AAPNet [15]. The former is a pyramidal neural network (i.e. a neural network with 2-D layers connected in cascade where each layer is smaller than the previous layer) with lateral inhibition that showed good results for dichotomous problems, such as face detection and forest detection. The latter is an autoassociative pyramidal neural network for one-class classification without lateral inhibition that achieved good recognition rates in the multiclass task of object categorization.

LIPNet, AAPNet, and other neural networks presented in the literature that use at least one of the discussed biological concepts (receptive fields, lateral inhibition, autoassociative memory) share the same problem: the configuration of the model have to be defined prior to the learning step generating a neural network with a static structure. The learning procedure of such neural networks are then restricted to the changing of the synaptic connections weights and dependent on knowledge from a specialist for the prior architecture configuration. Constructive learning [16] algorithm can overcome these limitations including new neurons or connections in the neural network topology as a function of the learning process. Ma and Khorasani [17] proposed a model with a constructive feedforward neural network for facial expression recognition. Additionally, a pruning step [18] was performed to reduce the size of the architecture without sacrificing the performance of the neural network. The technique proposed by Ma and Khorasani outperforms the methods using fixed neural network structure in computational efficiency, generalization and recognition performance capability.

In this paper, we propose the Constructive Autoassociative Neural Network (CANet) that incorporates the concepts of receptive fields, autoassociative memory, and lateral inhibition. The receptive field concept is used for implicit feature extraction preserving the spatial topology of the extracted features. Implicit feature extraction [19] enables the neural network to learn patterns from raw data integrating feature extraction and pattern classification in the same structure. On the other hand, the autoassociative memory is applied to reconstruct the input image with the features implicitly extracted from the raw image by CANet. A constructive-pruning algorithm dynamically updates the neural network architecture during the learning process in order to reduce the difference between the input image and the output of the CANet. The lateral inhibition in the output layer of CANet improves the recognition capability of the neural network [7], [13]. The parameters for the lateral inhibition are experimentally defined in this work.

### Definitions and Related Works

#### Receptive and inhibitory fields

Receptive fields concept refers to an area in which the presence of an appropriate stimulus produces response in a sensitive neuron and it was already identified in the visual, auditory and somatosensory systems of the human brain [2]. On the other hand, the inhibitory fields correspond to a region surrounding a given neuron that sends inhibitory stimulus simultaneously to the excitatory effect of the receptive field.

The concepts of receptive and inhibitory fields have already been used in image processing to improve texture analysis and contour detection accuracy [20]–[22], while ANNs inspired by such concepts have been proposed to incorporate feature extraction in their architecture for visual pattern recognition tasks [8]–[10], [23], [24]. In another work, we presented a study about receptive fields and lateral inhibition and proposed a pyramidal neural network for image classification that integrates both concepts, called LIPNet [7]. This neural network obtained low error rates, fast performance and low memory consumption.

#### Autoassociative memory

Autoassocative Artificial Neural Networks (AANNs) have the advantage of allowing non-linear correlations [25]. AANNs are based on autoassociative memory that is a type of memory where the input pattern and the desired output are the same. The classifiers based on such memory are useful to determine whether or not a pattern is known. AANNs have been successfully applied in many computer vision tasks. Thompson et al. [26] applied AANNs for novelty detection demonstrating that the learning is more ample than a simple memorization. Cavalcanti et al. [27] applied an AANN in a face verification problem. Wang and Chen [28] proposed an autoassociative model, called EFMAM, to perform pattern recognition tasks obtaining improvements in comparison with the not autoassociative version of the proposed model. Rao et al. [29] performed emotion recognition in image sequences by extracting features from face regions with a five layers AANN.

The output of the AANNs is the reconstruction of the input pattern presented to them. They have a bottleneck structure with fewer neurons in the hidden layers, responsible for data compression, than in the input and output layers. The pattern is mapped to a new feature space in the hidden layers and then the neural network learn the inverse mapping with respect to the minimization of the distance from the input to the output pattern. The AANNs have the ability to implicitly select and extract the features of the input data without any *a priori* knowledge or specific instruction [27]. However, this kind of representation is likely to have a high computational complexity due to the high number of neurons and synaptic connections.

AAPNet [15] is an autoassociative pyramidal neural network that achieved good results in object categorization. This neural network has a pyramidal architecture composed of receptive fields and shared weights reducing the number of synaptic connections between the neurons.

#### Constructive neural networks

Constructive algorithms [30] are learning methods used to adaptively adjust the architectural models of the ANNs. Many algorithms have been proposed to update the architecture of a neural network [31], such as:

- Constructive: add layers, neurons and connections to provide a minimal neural network architecture during the training.
- Pruning: remove layers, neurons and connections that are redundant from a neural network with a larger and deeper structure during the training.
- Constructive-Pruning: hybrid approach in which the neural network is pruned after a constructive process.
- Regularization: add or remove a punish term to the error function for discard not important connections during the training [32].

Sharma and Chandra [31] and Kwok and Yeung [33] presented a literature review for the constructive algorithms, emphasizing two approaches: Cascade-Correlation (CC) and Dynamic Node Creation (DNC). The CC algorithm [34] creates neural networks with multiple hidden layers with one neuron each that is connected to all other neurons previously added. This algorithm enables the neural network to detect high order features in the input pattern. However, the neural network generalization ability decreases as the number of the neurons added increases and the stimulus propagation might become very slow [33]. CC expansions have been proposed to allow, for example, more than one neuron in a same layer, but the decision about in which layer a new neuron should be added is not trivial and algorithms like the proposed by Ma and Khorasani [35] and Islam et al. [36] have been used limiting the number of neurons that can be added in each layer.

DNC is a model proposed by Ash [37] to dynamically add neurons in a hidden layer until the neural network reaches an approximation of the precision of its output. This algorithm creates neural networks with one hidden layer training the entire neural network every time a new neuron is added. DNC is a simple algorithm which follows the convergence properties of the universal approximators [38] of the underlying architecture. The main disadvantage of DNC is that the search space is too large, increasing the computational cost and the convergence time [39].

The constructive algorithm of an one-hidden-layer feedforward neural network (OHL-FNN) [33] is an extesion of the DNC algorithm used to avoid high computational cost. OHL-FNN freezes the neural network weights that have been previously trained and with an addition of a new neuron, the weights affected by the insertion are retrained. Ma and Khorasani [17] used the OHL-FNN strategy for facial expression recognition achieving a better classification rate than other neural network models with fixed structure.

In problems of one-class classification with many patterns, like facial recognition, constructive learning is particularly interesting since each neural network is evolved to individually learn each expression. The main advantages of using constructive methods are the following [31], [40]:

- The model becomes more flexible allowing a search in the space of possible configurations of the neural network.
- The initial configuration of the model is easily defined because it should be as simple as possible.
- If the constructive algorithm is successful, the obtained neural network can be used to estimate the complexity of the learned problem.
- It is possible to incorporate domain specific knowledge in the neural network that can be modified with the emergence of new training patterns.
- A different neural network configuration can automatically be defined for each learned pattern instead of using the same predefined neural network architecture for all known patterns.

## Constructive Autoassociative Neural Network

The CANet is a model designed to implicitly extract features in a dynamic architecture aiming to reconstruct the input image in the output layer that belongs to the OHL-FNN [33] approach. It uses the concepts of receptive fields and autoassociative memory to represent a visual pattern with implicit feature extraction in an one-class classification model. In order to optimize the architectural configuration for each known pattern, a constructive algorithm for the neural networks with one hidden layer is used, this choice was motivated by the work of Ma e Khorasani [17].

**Figure 1:**
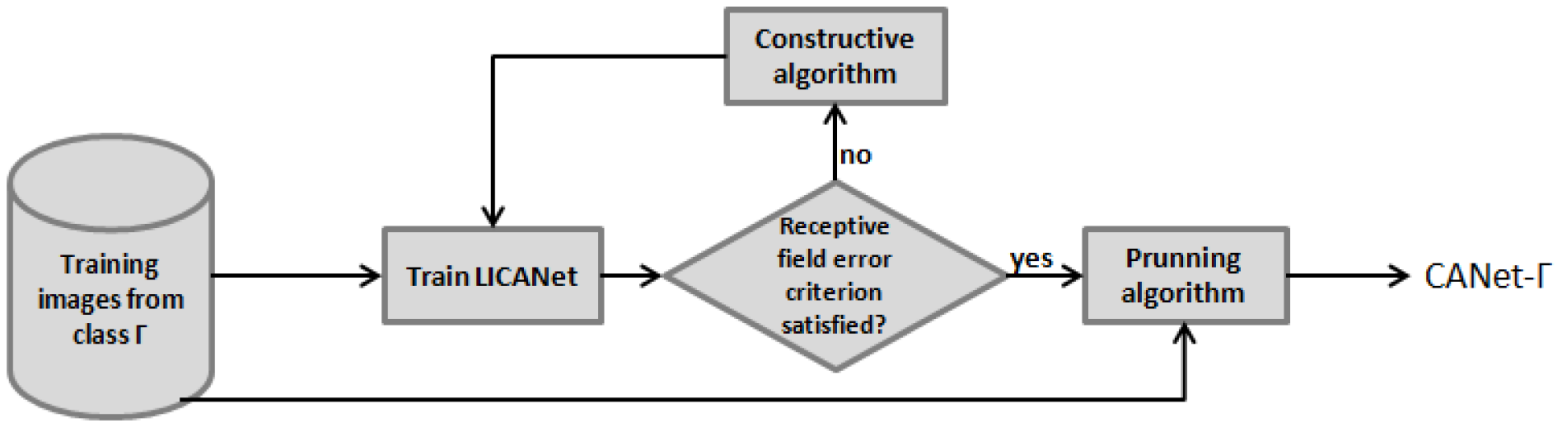
CANet training model for a set of training images from the class 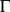. CANet-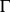 is the output of the model.

Fig. 1 presents the CANet training model. In each new training iteration, the learning algorithm evaluates if it should change the neural network architecture to improve the reconstruction accuracy. New neurons are added to the hidden layer in order to approximate the neural network output to the input image. It is important to note that each neuron in the hidden layer is connected to receptive fields in the same location of the input and output layers without any overlap between adjacent receptive fields. Thus, since neurons in the output layer do not have a bias connection, all the neurons in the same receptive field region return the same value. In order to avoid noisy pixels in the reconstructed image that could impair the image classification, a pruning algorithm [18] eliminates the neurons in the output layer that present the highest error rates in the training set, reducing the computational cost of the CANet without compromising its efficacy.

In this section, we present the neural network architecture, the connectivity model, the training algorithm, the constructive and prunning algorithms and the multi-class recognition system. Table 1 presents the notation and definitions used to describe CANet.

**Table 1:** Notations and definitions used to describe the CANet.

Symbol	Description
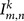	Value of the pixel in the 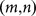 position of the k-th input image
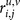	Receptive field size of the neuron 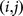 expanded from the neuron 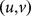
	of the CANet constructive layer
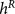	Size of the inhibitory field in the reconstruction layer
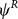	Strength of the lateral inhibition in the reconstruction layer
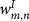 and 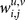	Weights associated with the position 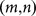 in the input layer 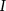 to the constructive
	layer and with the neuron 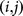 expanded from the neuron 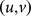
	of the constructive layer to the reconstruction layer, respectively
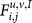 and 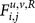	Receptive fields of the neuron 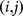 expanded from the neuron 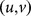
	of the constructive layer in the input and reconstruction layers, respectively
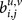	Bias of the neuron 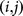 expanded from the neuron 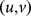 of the
	constructive layer
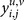 and 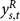	Outputs of the neuron 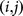 expanded from the neuron 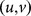 of the
	constructive layer and of the neuron 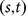 in the reconstruction layer
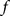	Activation function
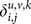 and 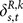	Error sensitivities for the neuron 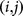 expanded from the neuron
	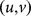 of the constructive layer and for the neuron 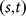 in the reconstruction
	layer 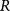 for an image 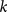 , respectively
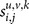 and 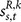	Weighted sum input for the neuron 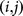 expanded from the neuron 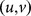
	of the constructive layer and for the neuron 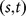 in the reconstruction layer
	for an image 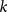 , respectively
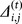	Adaption rule of the RPROP algorithm
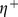 and 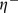	Increase and decrease factors of the RPROP algorithm
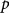	Maximum number of hidden neurons
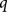	Number of neurons not removed by the pruning algorithm in the reconstruction layer
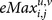 and	Maximum and minimum mean error rates of the neurons in the output layer contained
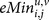	in the receptive field of the neuron 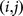 expanded from the neuron 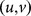
	of the constructive layer, respectively
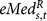	Mean error rate of the neuron 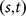 in the reconstruction layer
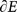	Error gradient

Notation and definitions used to describe the CANet.

### CANet Architecture

**Figure 2:**
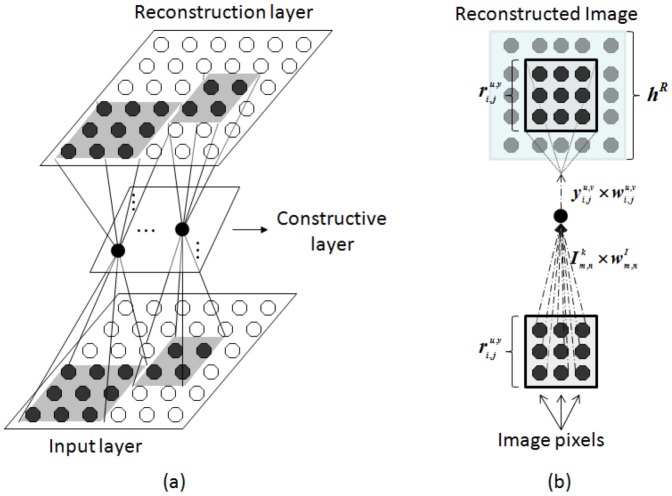
CANet architecture composed of 2-D layers in a bottleneck shape for image autoassociation: (a) network layers in which neurons are connected to receptive fields with different sizes in the input and output layers; (b) connectivity model of a neuron in the constructive layer.

Fig. 2 presents the CANet architecture that is composed of 2-D layers connected in cascade, *i.e.*, the output of one layer is the input to the next one. First, the input pattern is reduced to a feature map that is smaller than the input layer. The extracted features are then used to reconstruct the input image in the output layer.

The CANet architecture is composed of three layers:

- Input layer: each neuron in this layer represents a pixel in the input image and is associated with a weight 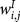. Thus, the images used as input to the neural network must have the same size of the input layer.
- Constructive layer: this layer is responsible to extract the features from the input image. New neurons are added in this layer during the training based on the error sensitivity of the neurons in a same receptive field of the reconstruction layer, 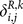, where 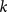 denotes a given training image.
- Reconstruction layer: responsible for returning the reconstruction of the input image using the features extracted in the constructive layer. Neurons in a same receptive field in the reconstruction layer share the same weight from the constructive layer.

The receptive field of a neuron is given by 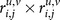 where 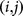 denotes the position of the neuron in the constructive layer that was previously generated from the neuron 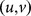. The output of a neuron in the constructive layer, 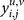, depends on the pixel value, 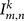, and on the weight associated to it, 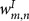, of all the pixels in the receptive field of the neuron. The constructive neuron output and the weight associated to it, 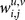, are used to reconstruct the input image along with the inhibitory stimulus sent by the neurons in the reconstruction layer with the size of the inhibitory field given by 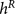. The output of the neuron in the reconstruction layer, 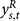, is the neural network output and it is an approximation of the pixel in the position 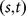 of the input image.

### Connectivity Model

The first layer of the CANet is the input image. The second layer is the constructive one. Each neuron in the constructive layer is associated to another neuron from which it was previously generated. The label of a neuron is given by two pairs of coordinates, the lower index 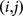 and the upper index 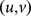. 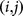 is the coordinate of the neuron after the division process, while 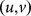 corresponds to the lower index coordinate of the previously divided neuron. The first neuron is located in position 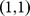 of the constructive layer and it is not associated with any neuron, represented by 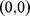. All the other neurons are generated from this neuron or from one of its descendants. Neurons in the constructive layer are connected to receptive fields with different sizes in the input and output layers.

The output of each neuron in the constructive layer consists in the application of a non-linear activation function over the weighted summation of the neurons in its receptive field. Thus, considering that 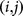 is the position of a neuron expanded from the neuron in the position 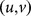 of the constructive layer, 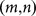 the position of a pixel in the input layer and 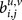 the bias associated to the neuron in position 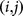, the output 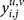 of the neuron in the constructive layer is given by 
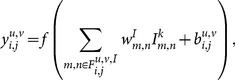
(1)where 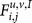 is the receptive field in the input layer of the neuron in the position 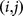 in the constructive layer.

The output of a neuron in the reconstruction layer, 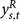, depends on the output of the neuron in the constructive layer that contains it in its receptive field, represented by 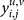 and on the lateral inhibition effect caused by neurons in its neighborhood. 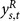 is calculated in three steps:

- Excitatory stimulus: for each neuron in the reconstruction layer, the excitatory stimulus is calculated using the following equation 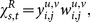
(2)where 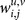 denotes the weight associated with the neuron in position 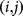 expanded from 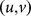 in the constructive layer that contains the neuron 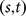 of the reconstruction layer in its receptive field, 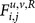.
- Inhibitory stimulus: for each neuron in the reconstruction layer, the lateral inhibition is calculated using the following equation 
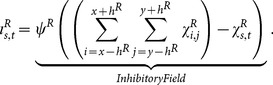
(3)
- Activation function: the excitatory and inhibitory stimulus are combined as input to a non-linear activation function that is monotonically increasing, continuous, differentiable and bounded, given by 
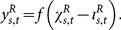
(4)

In this work, 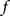 is the sigmoid function.

### CANet Training

CANet is a supervised neural network that tunes its weights during the training in order to reduce the error calculated through the output obtained and the input image. In each training step, first the error sensitivity for each neuron in CANet is calculated. Thus, the error gradients for the weights are derived. Finally, the weights are updated in order to learn a visual pattern with a pre-defined architecture configuration. The constructive algorithm that optimizes the CANet configuration is shown in the next section.

The error sensitivity 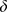 for each neuron in the reconstruction layer for an input image 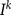 is calculated in three steps:

- Image error: difference from the obtained output and the pixel intensity, given by 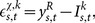
(5)where 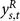 is the neuron output and 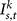 is the pixel intensity
- Sensitivity of the same layer: calculated using the image error summation of the neurons in the reconstruction layer that contains the neuron in the 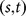 position in the inhibitory fields, given by 
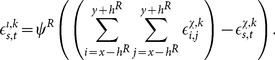
(6)
- Sensitivity of the neuron: 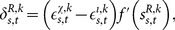
(7)where 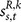 is the input for the neuron 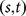 at the reconstruction layer, 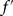 is the differential of the activation function 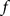 and 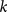 is the index representing each training image.

The error sensitivity for the neurons in the constructive layer is given by 
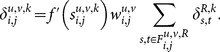
(8)


Furthermore, the error gradient, 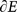, of the weights and the biases can be derived as follows.

- Error gradient of the weights in the constructive layer, 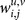:
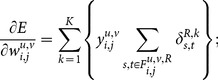
(9)
- Error gradient of the weights in the input layer, 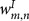, where 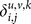 represent the error sensitivity of the neuron in the constructive layer that contains the neuron 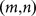 of the input layer in its receptive field:

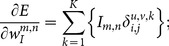
(10)
- Error gradient of the biases:
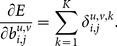
(11)

Finally, the weights in the neural network must be updated following a given learning rule. In this work, the Resilient Propagation (RProp) [41] is used. The RProp is known as an algorithm that converges fast with high accuracy and robustness [42]. The RProp updates the weights taking in account only the sign of the partial derivative over all patterns. Thus, the weights are adaptively updated based on the gradient signal, according to the following rule: 
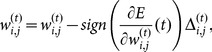
(12)and 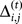 is the adaptation rule given by 
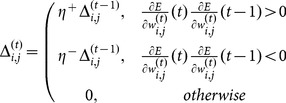
(13)where 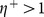 and 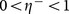 are the increase and decrease factors, respectively, that define the jump given in each learning step.

### Constructive and Pruning Algorithms

Constructive learning algorithms optimize the neural networks configurations for pattern classification without *ad hoc* choices [40]. On the other hand, pruning algorithms delete redundant connections in the neural network improving the efficiency of the model without compromising the effectiveness. Algorithm 1 in Table 2 presents the hybrid constructive-pruning algorithm proposed to train the CANet.

**Table 2:** Algorithm 1: Pseudocode of the constructive-prunning algorithm.

**Require:** 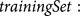 training images
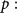 maximum number of neurons in the hidden layer
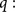 number of neurons not removed by the pruning algorithm in the reconstruction layer
**Ensure:** 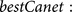 trained
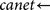 new CANet with one neuron in the hidden layer;
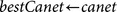
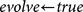 ;
** while** 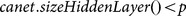 **do**
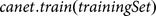 ;
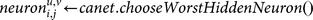
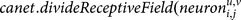 );
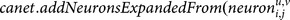 );
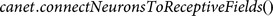 ;
** if** 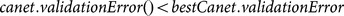 **then**
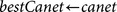 ;
** end if**
** end while**
** for all** 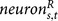 in 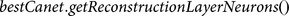 **do**
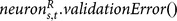 ;
** end for**
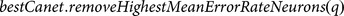 );

Algorithm 1: Pseudocode of the constructive-prunning algorithm.

Initially, there is only one neuron in the hidden layer with a receptive field containing all the neurons in the input and output layers and new neurons are iteratively added to the hidden layer. With the addition of each neuron in the hidden layer, the neural network is retrained by updating only the new connections. The criteria chosen to add new neurons in the hidden layer is based on the mean error rate for the receptive fields of the hidden neurons in the output layer. At each iteration, the neuron with the highest difference between the maximum and the minimum error values, 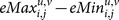, is expanded by dividing its receptive field in other four equal size receptive fields and three new neurons are added to the hidden layer.

**Figure 3:**
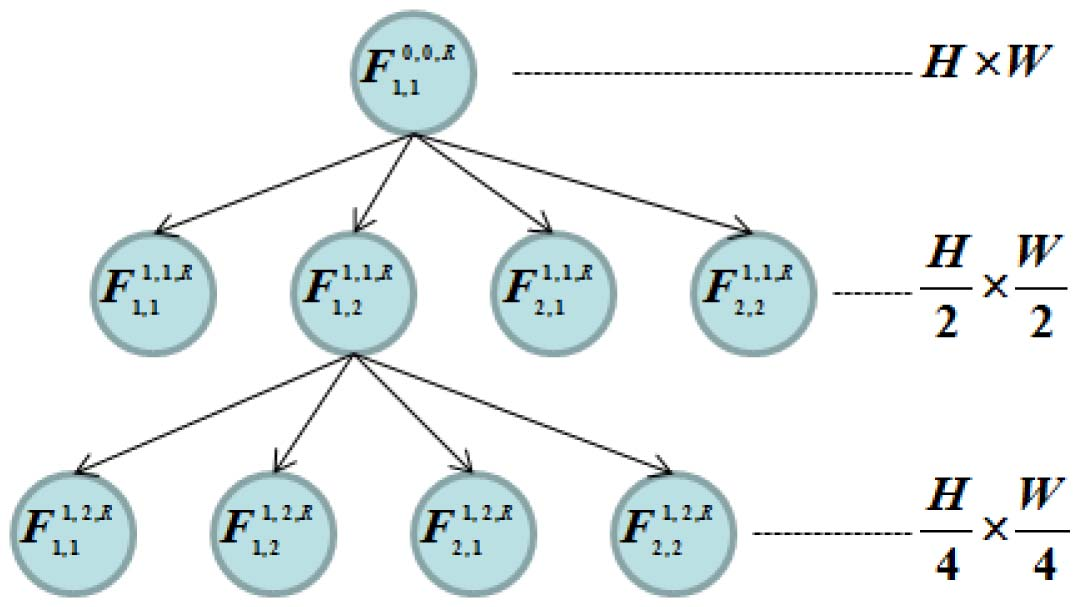
Quadtree model of the receptive fields hierarchy. Initially, there is only one receptive field with the same height and width of the output layer, given by 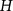 and 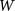, respectively. The receptive field is divided into four receptive fields with sizes 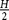 and 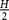. Finally, the receptive field denoted by 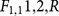 is divided into four receptive fields with sizes 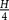 and 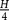.

The receptive fields are divided aiming to connect the neurons to homogeneous regions of the input image and they are indexed using a quadtree model. Fig. 3 presents this structure. Initially, there is only one receptive field with the same height and width of the output layer, given by 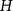 and 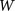, respectively. The receptive field is divided into four receptive fields with sizes 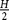 and 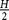. Finally, the receptive field denoted by 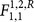 is divided into four receptive fields with sizes 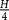 and 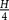. The quadtree model of CANet indicates how the homogeneous regions are distributed along the input patterns. After the expansion of the quadtree, each one of the new neurons are related to one of the generated receptive fields and the CANet is retrained. The receptive fields division process repeats until the maximum number of neurons in the hidden layer 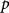 is achieved.

At the end of each expansion iteration, the validation error is calculated and the constructive algorithm returns the CANet with the lowest validation error disregarding the number of hidden neurons.

During the CANet training, it is possible that some neurons do not learn well the representation of some pixels. Thus, after the constructive training, a pruning step is performed in the CANet reconstruction layer and the neural network output is obtained considering only the 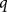 most similar pixels between the input image and the neural network output for all training images. The pruning algorithm keeps in the reconstruction layer only the neurons that approximates the neural network output to the input image with highest accuracy.

**Figure 4:**
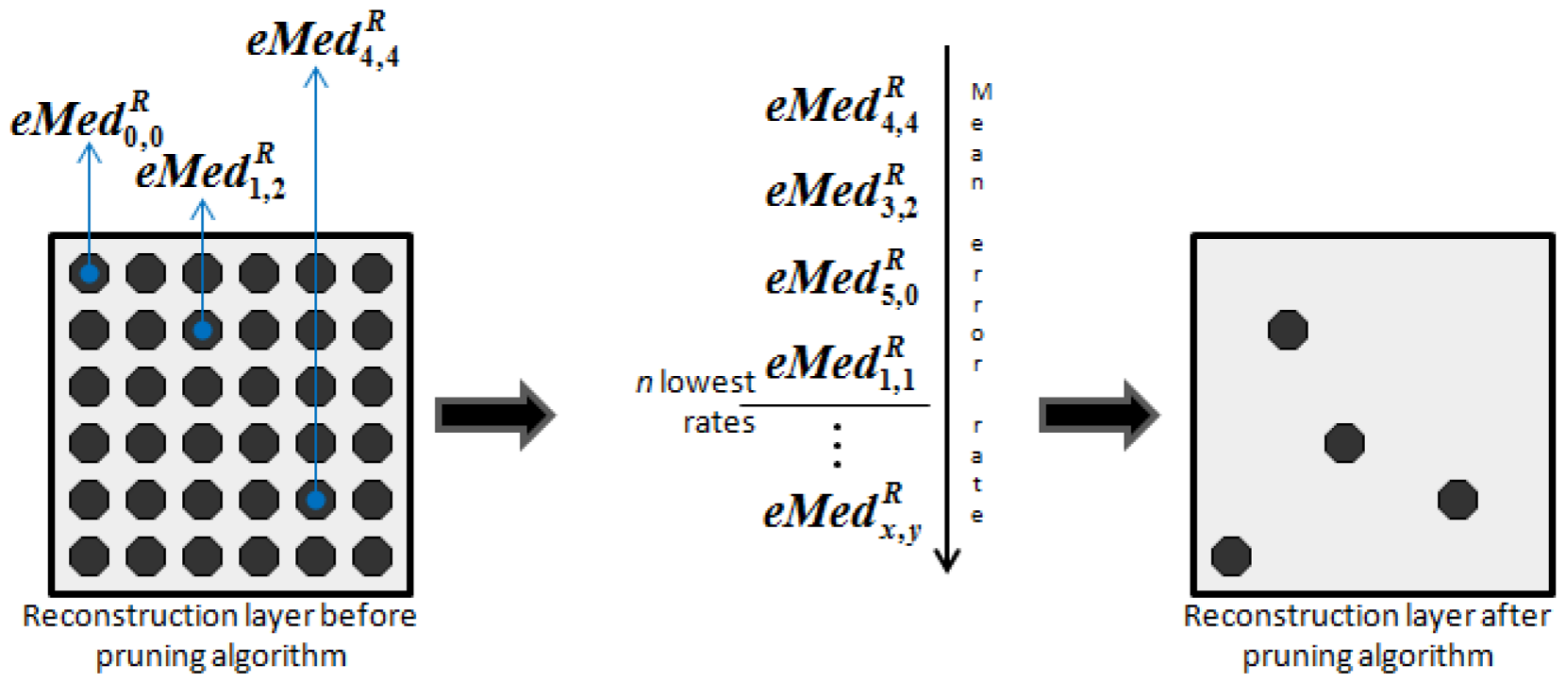
CANet pruning algorithm. The mean error rates are sorted and the neurons associated with the 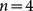 lowest rates are kept in the reconstruction layer.

Fig. 4 presents an illustration of the pruning algorithm. First, the mean error rate for each neuron in the reconstruction layer for all images used in the CANet training is calculated 
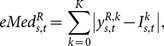
(14)where 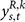 is the output of the neuron 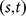 in the reconstruction layer for an input image 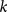, 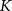 is the number of images used to train the CANet and 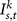 is the intensity of the pixel in position 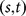 of the input image 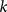. Second, the mean error rates are sorted and the 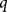 lowest mean error rates are selected. Finally, the neurons in the reconstruction layer associated with the selected rates are kept while the remaining neurons are removed.

### Multi-class Recognition System

CANet is a neural network for one-class learning. The CANet training defines a closed decision boundary and the distance from a pattern to such boundary is a measure of dissimilarity between the pattern and the class represented by the CANet. In multi-class tasks, a CANet committee is created for each class.

**Figure 5:**
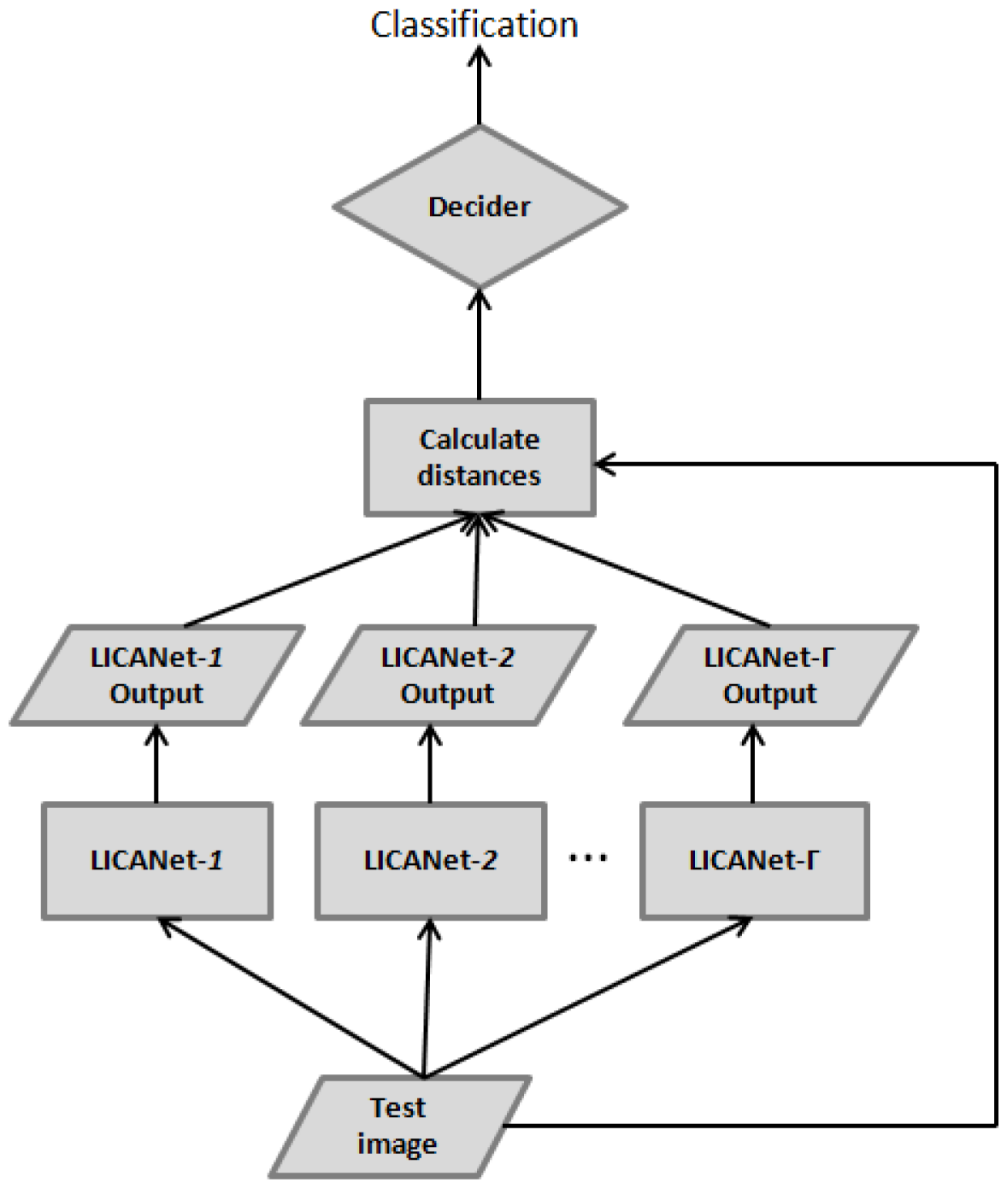
Multi-class recognition system of the CANet.

Fig. 5 presents the multi-class recognition system of the CANet. The test image is used as input to each trained CANet and a decider assigns the recognized class using the distances from the input image to the obtained outputs, given by 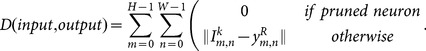
(15)


In this work, the classification is set using the minimum operator. Therefore, the committee output is the minimum distance obtained among all trained neural networks.

## Experiments

In this section, we compare the results obtained by the CANet with other methods in the literature in order to demonstrate the effectiveness of the proposed model. Three public databases were used: ORL [43] and AR [44] databases for face recognition; and JAFFE [45] for facial expression recognition. The experiments with the JAFFE database were also performed to determine the CANet parameters that were used in the face recognition experiments with ORL and AR databases.

### JAFFE Database

**Figure 6:**
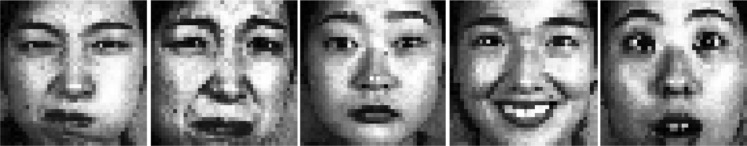
JAFFE images after pre-processing.

JAFFE (Japanese Female Facial Expression database) [45] was created to evaluate different methods for facial expression recognition [46]–[49]. JAFFE contains images from the six basic facial expressions plus the neutral expression, collected from 10 persons, presenting 3 or 4 images of each expression for each person. Experiments with the JAFFE database were performed using downsampled images leading to a lower computational cost. The original images are cropped to 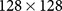 pixels to reduce the background influence. Cropped images are then scaled to 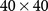 pixels with the histogram equalized. Feature extraction methods are not used and the pre-processing steps are independent of the image class. Fig. 6 presents some images after the pre-processing.

CANet has 4 parameters that have to be experimentally determined: maximum number of neurons in the hidden layer 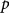, the number of neurons considered in the output layer 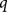, the size of the inhibitory field 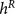 and the lateral inhibition strength 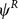. The following experiments show the influence of each parameter in CANet facial expression recognition using the first test approach with two randomly chosen images of each expression per person for training and the remaining for test.

**Figure 7:**
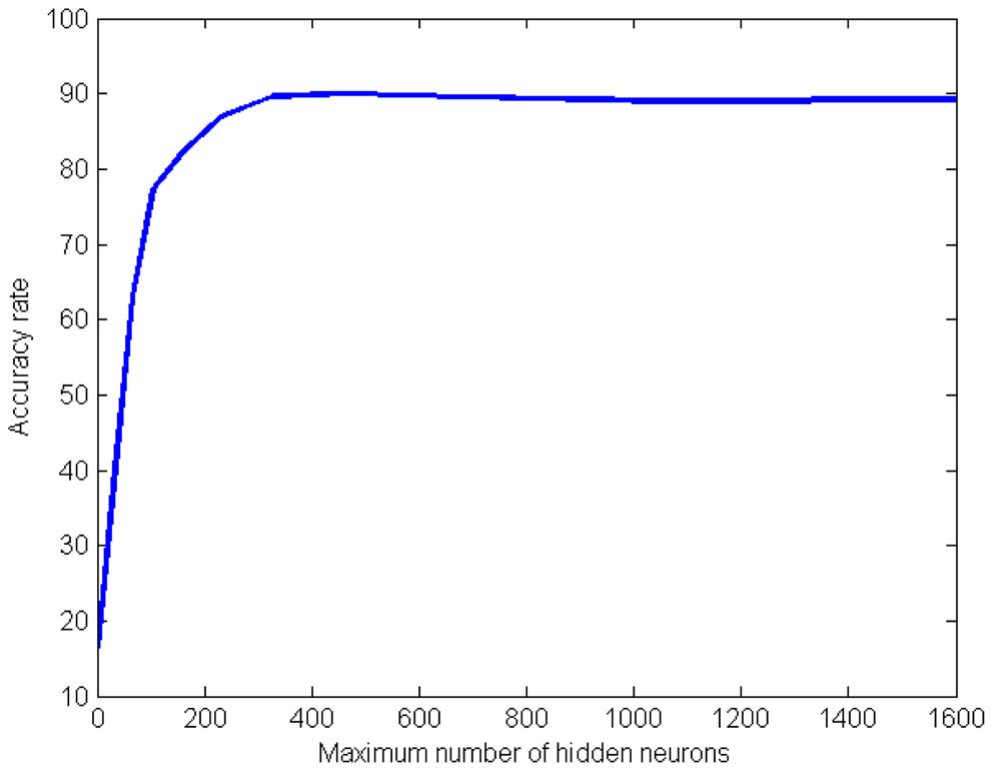
Facial expression recognition rate (%) for different values of maximum number of hidden neurons 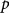.

The number of neurons in the hidden layer determines how sensitive the constructive algorithm is to the error. Fig. 7 presents the facial expression recognition rate for different numbers of neurons in the hidden layer using all the output neurons 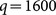, *i.e.*, no prunning is performed, and with no lateral inhibition. The highest recognition rate of 90.1% is achieved with approximately 25% of neurons in the hidden layer in comparison with the input size, such configuration is used in the following experiments.

The number of neurons considered in the output layer by the prunning algorithm, given by 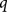, determines how sensitive is the CANet to variations in the learning for different image pixels, allowing the classification only with the pixels that better represent a class. The facial expression recognition rate is evaluated using only 50% of the neurons in the reconstruction layer that reaches a rate of 91.2%, being 1.1 percentile point higher than the rate obtained considering all the neurons in the CANet output. We experimentally evaluated other values for 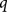, but they did not present improvements in the recognition. Thus, for the following experiments 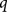 is set equal to 800.

Different configurations for lateral inhibition in the reconstruction layer of CANet are evaluated with the inhibitory field size and its strength varying from 1 to 10 and 1 to 25, respectively. Table 3 presents only the best results obtained varying the inhibitory field size, 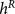, and strength, 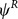. The highest recognition rate is obtained with the inhibitory size of 6 and strength equals to 17. Hypothesis test using t-Student with 5% of significance level between CANets with and without lateral inhibition statistically demonstrates that the presence of inhibitory fields improved the results obtained by the neural network.

**Table 3:** Facial expression recognition rate (%) for different configurations of inhibitory field size, 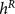, and inhibition strength, 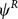, of the CANet in the JAFFE database.

Inhibitory configuration	Recognition rate (std)
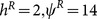	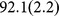
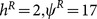	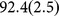
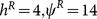	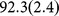
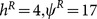	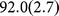
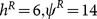	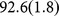
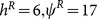	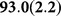
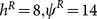	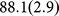
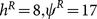	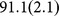

Facial expression recognition rate (%) for different configurations of inhibitory field size, 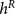, and inhibition strength, 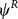, of the CANet in the JAFFE database

Two approaches are used to evaluate CANet in facial expression recognition using the JAFFE database in comparison to other methods in the literature. In the first approach, we use the same methodology applied by Zhi et al. [46] that randomly chooses two images of each expression per person for training and uses the remaining images for test. Bashyal and Venayagamoorthy [50] used a similar approach. In the second approach, called leave-one-image-out cross-validation, each image in the database is used to test in one iteration while the remaining images are used for training. Such approach was used by Cheng et al. [47]. Each approach was repeated 30 times and the average recognition rate is presented.

Table 4 presents a comparison between the CANet, the AAPNet [15] and different methods with feature extraction. The first approach is used and the feature extraction methods which CANet is compared with are the ones presented by Zhi et al. [46] and Bashyal and Venayagamoorthy [50]. The best method with feature extraction presents a facial expression recognition rate of 91.5%, while CANet presents a rate of 93.0% indicating that process of implicit feature extraction is able to generalize the CANet learning with no need of any prior feature extraction method. Also the CANet classification rate is 3.2 percentile points higher than the one obtained by AAPNet. Table 5 presents the confusion matrix obtained with CANet.

**Table 4:** Comparison between the facial expression recognition rate (%) obtained by CANet and different methods with feature extraction with the first test approach in the JAFFE database.

Method	Recognition rate (std)
CANet	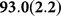
AAPNet [15]	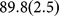
Gabor + LVQ [50]	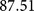
GSNMF [46]	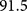
SNMF [46]	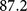
DNMF [46]	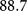
NMF [46]	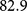
Laplacianfaces [46]	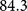
Fisherfaces [46]	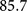
Eigenfaces [46]	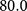

Comparison between the facial expression recognition rate (%) obtained by CANet and different methods with feature extraction with the first test approach in the JAFFE database.

**Table 5:** Confusion matrix of the CANet presenting the probability of an expression in the row to be classified as an expression in the column with the first test approach in the JAFFE database (SU: surprise, HA: happiness, AN: anger, DI: disgust, SA: sadness, NE: neutral, FE: fear).

	SU	HA	AN	DI	SA	NE	FE
SU	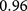	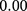	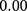	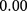	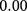	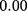	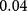
HA	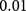	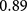	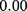	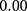	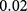	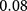	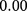
AN	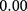	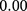	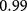	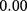	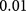	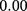	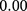
DI	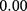	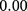	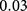	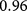	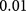	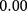	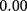
SA	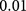	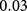	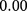	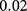	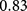	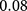	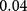
NE	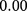	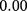	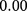	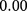	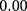	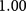	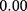
FE	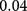	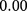	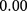	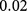	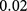	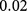	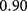

Confusion matrix of the CANet presenting the probability of an expression in the row to be classified as an expression in the column with the first test approach in the JAFFE database (SU: surprise, HA: happiness, AN: anger, DI: disgust, SA: sadness, NE: neutral, FE: fear).

In the second approach used to test the method, one image is used for test at each iteration while the remaining images are used for training. The results obtained by CANet with such approach are compared with other classifiers without feature extraction. Cheng et al. [47] proposed a gaussian classification process without any feature extraction method. Following the same approach, Cheng et al. [47] obtained a facial expression recognition rate of 93.4%. The CANet obtains a recognition rate of 99.9%. Table 6 presents the results obtained by the CANet, the AAPNet, the gaussian process and the k-NN classifier without feature extraction using the best value calculated for the neighborhood size. The autoassociative neural networks presents a recognition rate much higher than the other classifiers without feature extraction and the CANet presents the highest recognition rate.

**Table 6:** Comparison between the facial expression recognition rate (%) obtained by CANet and different methods without feature extraction with the second test approach in the JAFFE database.

Method	Recognition rate (std)
CANet	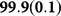
AAPNet [15]	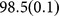
Gaussian process [47]	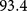
3-NN	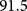

Comparison between the facial expression recognition rate (%) obtained by CANet and different methods without feature extraction with the second test approach in the JAFFE database.

### ORL Database

The ORL (Cambridge Olivetti Research Lab) face database [43] contains 400 different images, taken at different times from 40 people of different gender, age and race. The face images include variations in expression (such as open or closed eyes and smiling or not smiling), details (such as glasses/no glasses), rotation (up to about 20 degrees) and scale (up to about 10%). In this face recognition experiment, the main concern is to recognize thumbnail-sized face image, which requires less storage memory and recognition time. Thus, all the images were subsampled to 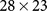 pixels.

Table 7 shows the comparison between the error rate obtained with the CANet and the results presented by Zhu et al. [51]. The CANet presented the lowest error rate in all the experiments using 3, 4 and 5 training images.

**Table 7:** Comparison between the error rate for face recognition (%) obtained by CANet and different methods from the work of Zhu et al. in the ORL database.

Method	Number of training images per class		
	3	4	5
CANet	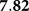	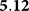	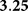
IMSEC [51]	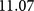	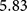	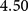
CMSE [51]	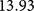	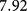	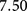
CRC [51]	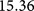	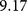	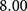
SRC [51]	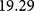	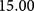	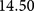
Eigenface [51]	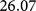	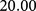	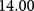
Fisherface [51]	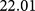	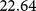	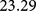
1-NN [51]	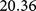	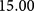	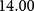
2DPCA [51]	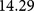	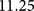	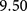
2DLDA [51]	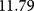	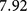	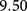

Comparison between the error rate for face recognition (%) obtained by CANet and different methods from the work of Zhu et al. in the ORL database.

### AR Database

The last experiment was conducted on the AR face database [44] using a cropped version with images from 50 males and 50 females [52] downsampled to 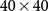 pixels. Twenty six images divided in two sessions were captured from each person. The same experiment protocol of Mi et al. [53], [54] was used and for each individual 7 images from the first session without any occlusion were used for training, while the 7 correspondent images from the second session was used for testing. Table 8 shows that the CANet achieves the highest recognition rate in comparison with the methods presented by Mi et al. [53], [54] such as Linear Regression-based Classification (LRC), Robust Linear Regression-based Classification (CLRC) and Sparse Representation-based Classification on K-Nearest Subspace (SRC-KNS).

**Table 8:** Comparison between the face recognition rate (%) obtained by CANet and different methods from the works of Mi et al. in the AR database.

Method	Recognition rate
CANet	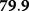
RLRC 1 [53]	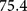
RLRC 2 [53]	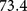
LRC [53]	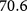
SRC-KNS [54]	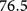

Comparison between the face recognition rate (%) obtained by CANet and different methods from the works of Mi et al. in the AR database.

## Discussion

In this paper, we proposed a novel neural network inspired by biological concepts present in the brain, called CANet. The proposed model is a constructive autoassociative neural network that returns as output an approximation of the presented input image using a dynamic architecture. CANet presents the concepts of receptive fields for implicit feature extraction and lateral inhibition and autoassociative memory for image reconstruction.

The CANet is an one-class classifier that connect the hidden neurons to homogenous regions in both input and output layers of the neural network. A constructive algorithm is applied in order to find the number of neurons in the hidden layer that minimizes the mean distance between the input images and the neural network outputs. After the CANet training, a prunning algorithm is used to keep in the output layer only the neurons with the highest accuracy in the training set, improving both the classification accuracy and the computational cost. It is important to note that the constructive algorithm improves the implicit feature extraction process performed in the hidden layer, while the pruning algorithm removes the redundancy of the output layer. Finally, the recognition system presented allows the use of the CANet in multi-class tasks. We showed that CANet outperforms other state-of-the-art algorithms in facial recognition tasks.

Improvements in the CANet could be applied, such as to create an overlapped region among adjacent receptive fields and to define different shapes for the receptive and inhibitory fields. In this way, allowing more hidden layers, which creates a deeper model. It is also interesting the possibility of using evolutionary techniques to find the most suitable architecture for the neural network. Moreover, experiments with other tasks rather than facial recognition should also be addressed.
